# Supplementary material for: HIVprotI: an integrated web based platform for prediction and design of HIV proteins inhibitors
Source: J Cheminform. 2018 Mar 9;10:12. doi: 10.1186/s13321-018-0266-y (PMC5845081; doi:10.1186/s13321-018-0266-y)
Supplement: Supplementary file 2 — Additional file 2. Source code of HIVProtI web server. [file 13321_2018_266_MOESM2_ESM.zip › HIVprotI_Source-code/hivprotI/jsmol/dssr.htm]

Jmol/DSSR Demonatration Page


|  |  |  |
| --- | --- | --- |
| This page demonstrates capabilities in Jmol 14.2 that involve RNA and DNA secondary structure using real-time DSSR analysis provided by 3DNA.     The commands being issued by the buttons are displayed in the box under the applet. |  |  |
|  |  |  |
